# Prescription Department

**Published:** 1893-11

**Authors:** 


FOR LEUCORRHŒA.

℞ Tr. Eucalypti, ℥j.

Dioviburnia, ℥vij.

M. Sig. Dessert spoonful three times a day.

INFANTILE COLIC.

Hare (*Col. and Clin. Rec.*) recommends the following in infantile colic:

℞ Sodii bromidi, gr. xlviij—xcvi.
Chloralis, gr. xxiv—xlviij
Syr. lactricarii, q. s. ad., f ℥iij.

M.

FOR WHOOPING COUGH.

℞ Bromoform, gr. ij.
Neurosine, ℥iv.

M. Sig. Shake and give a teaspoonful four times a day.

EPIDIDYMITIS.

For Epididymitis, Professor Steer recommends a formula like the following:

℞ Morph. sulph., gr. j.

Chloral hydrat., ʒiss.

Potas. bromid., ʒij.

Syrup., f ʒiv.

Aquæ, f ℥iiss.

M. Sig. Tablespoonful every three hours if necessary.—*Medical Fortnightly.*

GRANULAR LIDS.

Dr. G. Sterling Ryerson orders, applied at night:

℞ Hydrarg. oxid. flav., gr. iv.
Zinci oxid., gr. ij.
Thymol., M., ij
Camphor, gr. ss.
Cocain., muriat., gr. ij
Vaselin, ℥j.—M.

—*Therapeutic Gazette.*

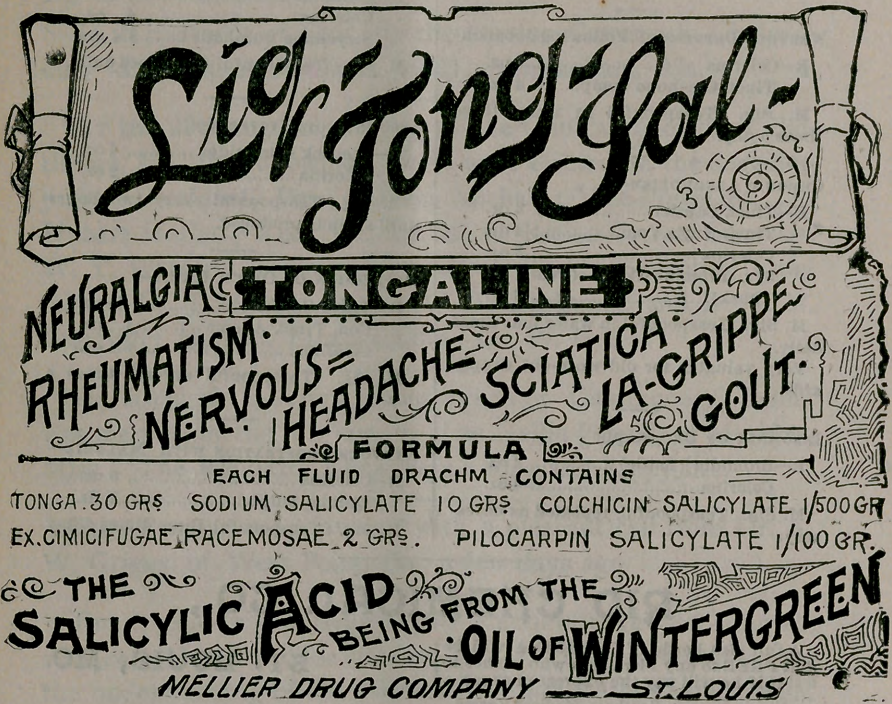

Please mention Southern Medical Record.

STUBBORN NEURALGIA.

℞ Arsenici iodid., gr. j.
Ext. belladon.,
Morph. valerian, aa gr. viij.
Pulv. ext. gentian., gr. v.
Ext. rad. aconit., M., v.
M. et ft. pil. no. xl.
Sig. One, two, or three every twenty-four hours.—*Medical and Surgical Reporter*.

A PLEASANT PURGATIVE.

Every physician has felt the want of a pleasant purgative for children. Dr. C. H. Smith writes to the Columbus Medical Journal that the following formula has been used by him for years:

℞ Glycerini ʒi.

Ol. Cinnamoni gtt. vi.

Trit. bene et adde

Ol. ricini ℥i.

M. S. ʒi. at a dose p. r. n.

CHRONIC CONSTIPATION.

For chronic constipation Dr. Delafield prescribes the following pill:

℞ Strychniæ sulph gr. 1-40.
Pulv. ipecac.
Ext. belladonna.
Ext. colocynth. Co. aa gr. 1-4.
M. Ft. pil. No. i.
S. Four pills daily at first; then three and afterwards two.

GONORRHŒA.

In any stage, try internally:

℞ Potassii bromidi, ʒiv.

Sodii Bicarbonatis, ℥j.

Tiuct. Cannabis Indicæ, ʒ iv.

Sts. Æth. Nitrosi, ℥iij.

Aquæ ad., ℥vj.

M. ft. sol. Sig. One drachm three times per day.

And as an injection:

℞ Ext. Pinus Canadensis (white) ℥ij.
Tinct. Opii, ℥jss.
Gylcerini, ℥jss.
Aquæ Rosæ, ad ℥vj.
M. Sig. Inject every three hours.

